# Effects of Chronic Consumption of Sugar-Enriched Diets on Brain Metabolism and Insulin Sensitivity in Adult Yucatan Minipigs

**DOI:** 10.1371/journal.pone.0161228

**Published:** 2016-09-01

**Authors:** Melissa Ochoa, Charles-Henri Malbert, Paul Meurice, David Val-Laillet

**Affiliations:** 1 UR1341 ADNC, Institut National de la Recherche Agronomique, Saint-Gilles, France; 2 US1395 Ani-Scans, Institut National de la Recherche Agronomique, Saint-Gilles, France; Chi-Mei Medical Center, TAIWAN

## Abstract

Excessive sugar intake might increase the risk to develop eating disorders *via* an altered reward circuitry, but it remains unknown whether different sugar sources induce different neural effects and whether these effects are dependent from body weight. Therefore, we compared the effects of three high-fat and isocaloric diets varying only in their carbohydrate sources on brain activity of reward-related regions, and assessed whether brain activity is dependent on insulin sensitivity. Twenty-four minipigs underwent ^18^FDG PET brain imaging following 7-month intake of high-fat diets of which 20% in dry matter weight (36.3% of metabolisable energy) was provided by starch, glucose or fructose (n = 8 per diet). Animals were then subjected to a euglycemic hyperinsulinemic clamp to determine peripheral insulin sensitivity. After a 7-month diet treatment, all groups had substantial increases in body weight (from 36.02±0.85 to 63.33±0.81 kg; *P*<0.0001), regardless of the diet. All groups presented similar insulin sensitivity index (ISI = 1.39±0.10 mL·min^-1^**·**μUI·kg). Compared to starch, chronic exposure to fructose and glucose induced bilateral brain activations, *i*.*e*. increased basal cerebral glucose metabolism, in several reward-related brain regions including the anterior and dorsolateral prefrontal cortex, the orbitofrontal cortex, the anterior cingulate cortex, the caudate and putamen. The lack of differences in insulin sensitivity index and body weight suggests that the observed differences in basal brain glucose metabolism are not related to differences in peripheral insulin sensitivity and weight gain. The differences in basal brain metabolism in reward-related brain areas suggest the onset of cerebral functional alterations induced by chronic consumption of dietary sugars. Further studies should explore the underlying mechanisms, such as the availability of intestinal and brain sugar transporter, or the appearance of addictive-like behavioral correlates of these brain functional characteristics.

## Introduction

Dietary sugar intake, in the form of sucrose or high-fructose corn syrup (HFCS), has dramatically increased and correlates with a rise in obesity, metabolic syndrome and type 2 diabetes [1]. In particular, fructose consumption has incrementally doubled in the last 30 years [2, 3] in part because of a partial substitution of sucrose (composed of 50% glucose and 50% fructose) by HFCS (47–65% fructose, 53–35% glucose) [4]. However, since fructose and glucose intake may vary simultaneously in the diet, the respective contributions of glucose and fructose in the development of metabolic and central abnormalities remain controversial [5]. We recently showed that chronic consumption of a fructose-containing diet induced a strong preference and motivation for this diet, while chronic glucose and starch intake did not increase over time the preference and motivation for these diets, respectively [6]. We attributed this fructose—induced fructose preference to an enhancement of fructose palatability and sweeter taste perception compared to glucose [7] in fructose-fed pigs, and we hypothesized a modification in the function of the brain reward circuitry.

Reward from eating is controlled by the mesolimbic dopamine (DA) pathway, which consists of dopaminergic cell bodies that reside in the ventral tegmental area and its projections to circuits implicated in reward, such as the insula, thalamus, striatum, amygdala, ventromedial prefrontal cortex, and orbitofrontal cortex (OFC) [8–10]. The dorsolateral prefrontal cortex (DLPFC) plays a role in the central regulation of eating behavior by inhibiting orexigenic areas to suppress hunger and terminate feeding [11, 12]. Food addiction scores correlated with activation in the anterior cingulate cortex, medial OFC, amygdala, DLPFC and caudate in response to chocolate milkshake intake [13], similar to neural activation patterns observed in addictive-like eating behavior and substance dependence [14]. A decreased activity in the OFC, DLPFC, and anterior cingulate cortex was associated with a reduction in dopamine type-2 receptor (D2R) availability as measured by positron emission tomography (PET) with ^11^C-raclopride [15, 16]. Interestingly, Volkow et al. [17] demonstrated a negative association between body mass index (BMI) and brain metabolism in the DLPFC. Stice et al. [18] also showed that women who gained weight over a 6-month period showed a reduction in striatal response to palatable food consumption relative to weight-stable women. Overall, body weight variations clearly have an impact on mesolimbic areas functioning, but are specific nutrients or diets able to have similar effects independently from body weight?

Several studies highlighted some differences in brain activation induced by glucose and fructose intake: glucose, but not fructose, decreased regional cerebral blood flow in reward and appetite regions (i.e., hypothalamus, insula, thalamus, and striatum) [19]. Besides, it was suggested that fructose-containing diets may lead to a reduced reward value from food [20] and might alter the capacity to perceive food reward and pleasure thereby altering motivation for fructose [2]. Smaller increases of insulin and leptin secretion induced by fructose intake, compared to glucose [21, 22] might contribute to this phenomenon. By promoting reduced insulin sensitivity and basal hyperinsulinemia [23–25], chronic fructose ingestion may alter dopamine neurotransmission, activity of brain reward regions, the hedonic response to food, and may drive excessive energy intake and modifications of eating behavior probably through a blunted reward response [2]. Consumption of a diet containing dietary fat and fructose, but not sucrose, resulted in a specific reduction of D2R signaling in rats’ brain, indicating that sucrose and fructose intake are regulated differentially by the mesolimbic system [26], or that these dietary sugars differentially affect reward systems including the dopaminergic system. Taking these data together, we suggested that the development of a fructose-induced fructose preference observed in our minipigs [6] might be related to modifications of the brain reward circuit and/or to differential insulin sensitivity, compared to glucose-fed animals. To our knowledge, no study has explored the long-term effect of different dietary sugars on changes in the activity of brain regions involved in reward and eating behavior and its association with insulin sensitivity in an animal model relevant to human nutrition and neurosciences, such as the minipig [27–30]. The (mini)pig model is increasingly used in biomedical research, and especially in nutrition and neurosciences studies. Similarities with the human in terms of cognition, development of food preferences and eating disorders, digestive anatomy and functions, as well as brain development and processes have been extensively described in recent reviews [5, 27, 31, 32]. Moreover, our group has previously documented the consequences of diet-induced obesity in the minipig in terms of eating behavior [33], physiology [29] and brain metabolism, highlighting for the first time ever that morbidly obese minipigs have similar brain anomalies as those described in humans, including a decreased metabolism in the prefrontal cortex and striatum in comparison to normal-weight subjects [30].

The aim of this study was to investigate the effects of chronic intake of high-fat isocaloric diets varying in a substantial fraction of their carbohydrate content: starch as control, glucose or fructose, on the activity of brain regions involved in reward (*i*.*e*., anterior prefrontal cortex—APFC, DLPFC, OFC, caudate nucleus, putamen, nucleus accumbens, and globus pallidus) measured by PET with ^18^F-fluorodeoxyglucose (^18^FDG). We explored whether these modifications in brain activity, in animals of similar body weight, could be related to peripheral insulin sensitivity using the euglycemic hyperinsulinemic clamp technique. We hypothesized that (1) chronic intake of glucose and fructose diets would provoke modifications in the activity of brain reward-related regions, compared to starch chronic consumption; (2) animals fed the glucose and fructose diets would present different insulin sensitivity compared to the starch group, independently of weight gain, and (3) chronic intake of fructose would induce more pronounced central and peripheral metabolic alterations than glucose.

## Materials and Methods

### Animals and diets

The present study was conducted in accordance with the current ethical standards of the European and French legislation (Agreement number A35-622 and Authorization number 01894 and 3588). Local Ethics Committee approved the entire protocol (R-2011-MO-01). Trained staff members provided animal care and management under the supervision of a veterinarian. Surgery was performed under isoflurane anesthesia, and all efforts were made to minimize suffering. A total of 24 adult Yucatan minipigs (12 males and 12 females; 12 to 14 months of age) (INRA, Saint-Gilles France), with initial weight of 36.6±1.0 kg before the beginning of the dietary intervention, were used in this study. Animals were housed in individual cages (150x60x80 cm) under controlled temperature (24°C), and maintained on a 12 hour light-dark cycle with free access to water.

Experimental diets compositions are shown in Table 1. Animals were fed daily at 8.00 h with 1 kg of pelleted standard minipig diet. Refusals (if any) were withdrawn and weighed at 16.00 h. Animals were randomly divided into three groups. Each group (n = 8 per group) received during 7 months one of three isocaloric high-fat diets, varying only in their carbohydrate sources: starch as control, glucose, and fructose. Animals received and consumed 1 kg of feed per day. This amount of feed represents approximately a 36% excess of the nutrient requirements in minipigs (10.31 MJ/kg, data from INRA St-Gilles, France), allowing for significant weight gain, but represents less than the amount animals could eat spontaneously and daily. This feeding plan was chosen to homogenize body weight gain between animals and groups, and to prevent morbid obesity that would be accompanied by comorbidities such as type-2 diabetes and cardiovascular diseases. Animals were weighed every two weeks during the whole experiment, before the beginning of the brain imaging and before the euglycemic-hyperinsulinemic clamp.

**Table 1:** Composition of standard and experimental diets for minipigs.

Composition (%)	Standard	Starch	Glucose	Fructose
Wheat	10.0	6.0	6.0	6.0
Barley	33.0	12.0	12.0	12.0
Wheat bran	25.0	14.0	14.0	14.0
Soybean meal	6.0	9.0	9.0	9.0
Sunflower meal	10.0	8.0	8.0	8.0
Soybean hulls	12.0	11.0	11.0	11.0
Sucrose	1.0			
Corn starch		25.0	5.1	6.5
Glucose source ^a^			19.9	
Fructose source ^a^				18.5
Lard	0.0	12.0	12.0	12.0
Dicalcium phosphate	0.6	0.6	0.6	0.6
Calcium carbonate	1.3	1.3	1.3	1.3
Salt	0.6	0.6	0.6	0.6
Oligoelements and vitamins	0.5	0.5	0.5	0.5
**Nutritional value (calculated** ^b^**)**				
Metabolizable energy (MJ/kg)	10.3	14.1	14.1	14.1
Net energy (MJ/kg)	7.3	10.9	10.9	10.9
Dry matter (%)		89.6	89.6	89.6
Crude protein (%)	15.2	12.2	12.2	12.2
Crude fat (%)	2.2	13.5	13.5	13.5
Cellulose (%)	11.0	8.0	8.0	8.0
Minerals (%)	6.8	5.6	5.6	5.6

^a^ Pure glucose and fructose sources with water content of 9.5 and 0.5%, respectively, so they both provided exactly 20.05% of the diets’ dry matter.

^**b**^ Nutritional values were calculated upon the nutritional tables from Sauvant *et al*. [34].

### Anesthesia and surgery

Since circulating estrogens can influence weight regulation, insulin sensitivity and brain activity, an ovariectomy was performed in female minipigs to prevent any effect of the estrus period on data collection. Animals were fasted for 15–18 hours before the surgery and imaging session. Pre-anesthesia was induced with an intramuscular injection of ketamine (5.0 mg/kg; Rhône Merieux, Lyon, France). Suppression of the pharyngotracheal reflex was obtained by inhalation of isoflurane (3–5% v/v, Baxter, France) immediately before intubation. A surgical level of anesthesia was obtained with isoflurane (2–3% v/v; Minimal Alveolar Concentration 2.0) delivered by a mechanical ventilator; Oxygen fraction (FiO_2_) and tidal volume were adjusted so that spO_2_ was 98% or more and spCO_2_ measured by infrared capnometer (Armstrong; Armstrong capnometer, Gambo Engström, Bromma, Sweden) was <5%. Analgesia was obtained by an intravenous injection of a morphinic agent (Fentanyl 0.4 ml/min, Renaudin, Paris, France). The ovariectomy procedure was performed using celioscopy under constant abdominal pressure of 14 mmHg (CO_2_ insufflation). After surgery, animals received a unique dose of morphine sulfate (0.2 ml SC). Animals had two weeks to recover before brain imaging, during which a similar anesthesia procedure was used.

### Brain imaging procedure

To investigate the effects of chronic exposure to carbohydrate-containing diets on basal cerebral glucose metabolism (CGM), animals underwent brain-imaging sessions following 7-month intake of starch-, glucose- or fructose-containing diets in the basal condition. The brain imaging modality was PET after ^18^F-fluorodeoxyglucose (^18^FDG, IBA, France) injection.

#### Animal preparation

Anesthetized animals were placed on Head First Prone position and a venous catheter was inserted into their left ear in order to inject the radiolabeled compound. To minimize auditory and visual stimulations, animals’ ears and eyes were sealed with cotton and surgical tape, respectively. Animals were covered with a heating blanket to maintain body temperature at 37°C during the entire procedure. Glycaemia was measured (OneTouch, GlucoTouch Plus, Lifescan, Milan, Italy) from the venous catheter to make sure that it was below 6 mmol/L before the beginning of the procedure.

#### Image acquisition

PET scans were obtained on a whole body, high-resolution tomograph (Siemens/CTI ECAT, 962, HR+) using ^18^FDG as radioligand. To correct photon attenuation, transmission imaging was performed prior to emission scanning with three rotative sources containing ^68^Ge. A 40-min 3-dimensional (3D) emission scan was started 45 min after injection of 300 MBq of ^18^FDG using an axial FOV of 15.52 cm. Following scatter, dead time and random corrections, PET transaxial images were obtained by iterative reconstruction using a ramp filter (Kernel FWHM = 6 mm) providing 63 contiguous slices. Spatial resolution after reconstruction was 0.64 mm per pixel in the x- and y-axis and 2.42 mm per pixel in the z-axis. Pixel depth was encoded using Standardized Uptake Value (SUV) method.

#### Image processing and statistical analyses

Data were analyzed with Statistical Parametric Mapping (SPM8, Welcome Trust Center for Neuroimaging, London, IK) implemented in MATLAB 7.1 (The Mathworks Inc., Natick MA, USA) for spatial preprocessing and statistical analysis. PET images obtained in our study were reoriented, centered on a reference point (x_0_, y_0_, z_0_, posterior commissure), and then masked to remove the extra cerebral matter. Coordinates were realigned on a brain template from the pig atlas [35]. Images were then spatially normalized using the template created in the laboratory and smoothed with a 4x4x4-mm full width Gaussian filter.

In the whole-brain analysis, the regional ^18^FDG uptake was standardized to the mean global uptake of the entire brain using global scaling in order to minimize variations across animals’ brain in global CGM. Analyze of covariance (ANCOVA) was used in the SPM analyses in order to include the mean corrected global activity as an additional regressor in the model, which is adapted for our PET scan protocol with a controlled administered dose and allows correction for family-wise errors (FWE). Images from the starch, glucose, and fructose groups were compared using paired *t*-tests. Three contrasts (starch *vs*. glucose, starch *vs*. fructose, and glucose *vs*. fructose) were performed to determine the bidirectional differences of brain metabolism, *i*.*e*. both higher and lesser basal CGM responses of one treatment compared to another. A small volume correction (SVC) analysis was performed with SPM8 on the regions of interest (ROI) selected upon the *a priori* hypotheses. For this analysis, we selected the following structures (bilaterally) involved in food reward and regulation of eating behavior: APFC, DLPFC, OFC, caudate nucleus, putamen, nucleus accumbens, and globus pallidus, for which we hypothesized “a priori” a modulation in their activity induced by dietary sugars, specially fructose, compared to starch as control diet.

The statistical analysis with SPM8 produced a list of voxels for which the activation (CGM) differed between treatments. Each voxel was associated with a set of coordinates (x y z) corresponding to its spatial location in the CA-CP (*commissura anterior-commissura posterior*) plane with CP set as the origin. The ROIs chosen were anatomically identified on the basis of a 3D digitized pig brain atlas developed in our laboratory [35]. Then, voxels for which activity in brain structures was statistically different between dietary treatments were identified. A *P*-value uncorrected at *P* = 0.001 was set as the threshold (5 voxel extent threshold in uncorrected).

### Euglycemic-hyperinsulinemic clamp procedure

The clamp procedure was performed four weeks after PET imaging. After an overnight fast, minipigs were weighed and anesthetized with the same procedure as for PET imaging. Body temperature was maintained at 38.0±0.05°C by a self-regulating heating element placed under the animal. Animals were clamped at their individual fasting blood glucose concentrations (minimum 3.5 mmol/L). Immediately after pre-anesthesia with ketamine, animals were weighed in order to have their exact weight for clamp performance, and then anesthetized with isoflurane. Before the beginning of the clamp, one catheter was inserted in the carotid artery for blood sampling. A second catheter was inserted in the internal jugular vein for infusion of 30% glucose solution. Finally, a third catheter was inserted into the marginal ear vein for insulin perfusion. Two arterial blood samples were obtained 20 and 10 min before (T-10 and T-20 min) starting glucose and insulin infusions for glycaemia (One-Touch II; Lifescan, Roissy, France) and plasma insulin concentration measurements with a commercially available kit (Insulin-CT radioimmunoassay; MP Biomedicals, Orangeburg, NY, USA) using a gamma counter (Pharmacia-Wallac, Turku, Finland). The calculated mean glycaemia at T-20 and T-10 min was used as basal glycaemia for DeFronzo algorithm [36]. Insulin (Actrapid, 100 UI /ml, Novo Nordisk, Bagsvaerd, Danemark) was injected into the ear marginal vein catheter as a prime dose (50 mU/kg). Constant hyperinsulinemia was achieved by a continuous insulin perfusion (5 mU/kg/min; Perfusor secura FT; B. Braun). Arterial blood glucose concentration was assessed at 5-min intervals, which was introduced in the computer software running the euglycemic hyperinsulinemic clamp algorithm (Labview, Clamp 4.0 C.H. Malbert). The computer calculated, according to DeFronzo algorithm [36], every 5 min the amount of glucose necessary to maintain glycaemia at the basal level. This computer controlled a pump (Syringe infusion pump 22, Harvard Apparatus; Healing SARL, Les Ulis, France) to infuse the 30%-glucose solution (B. Braun Medical SA, Boulogne, France) into the internal jugular vein at the calculated variable rate to maintain euglycaemia. The euglycemic-hyperinsulinemic clamp was performed over a period of 180 min. The first 150 min were necessary to reach the steady state for arterial blood euglycemia. Arterial blood samples were obtained at T160, T170, and T180 min after the beginning of the procedure for glycaemia and plasma insulin concentration measurements. Insulin infusion was finished after 180 min. Animals were euthanized at the end of the procedure by T61 injection.

#### Calculations for euglycemic-hyperinsulinemic clamp data

Since there is no change in blood glucose concentrations under steady state clamp conditions and that the hyperinsulinemic state is sufficient to completely suppress the hepatic glucose production, the glucose infusion rate (GIR) must be equal to the glucose disposal rate (M), and constitutes an estimate of insulin sensitivity. The insulin sensitivity index (SI) derived from the clamp data was defined as: SIClamp = M / (G x ΔI)

Where M is the (GIR) (mg/min) adjusted to weight; G is the steady state blood glucose concentration (mmol/L), and ΔI is the difference between fasting and steady state plasma insulin concentrations. Steady-state glucose infusion rate (SSGIR) was determined as the GIR that guaranteed over a period of at least 30 min a constant glucose concentration on the basal level [37].

#### Statistical analyses of euglycemic-hyperinsulinemic clamp

All the statistical analyses were conducted using R 2.15.0 software (The R Foundation for Statistical Computing 2012).

Body weight (kg), glucose infusion rate (GIR, mg/min), GIR adjusted to weight (GIR/kg body weight), fasting and steady state plasma glucose concentrations (mmol/L), fasting (at min -10) and steady state plasma insulin concentrations (mUI/L), and the insulin sensitivity index (ISI) presented a Gaussian distribution. Clamp data were analyzed by a generalized linear model (glm) and two-way analysis of variance (ANOVA), with diet (starch, glucose or fructose) and sex as independent variables. Significant effects were further analyzed using pairwise comparisons with False Discovery Rate (FDR) correction. Results were presented as mean ± SEM. Differences were considered statistically significant if *P*<0.05.

## Results

As expected, 7 months of obesogenic diets induced a substantial increase in body weight (from 36.02 ± 0.85 to 63.33 ± 0.81 kg; *P* < 0.0001) regardless of dietary treatments. Clamp data and body weight are presented in Table 2.

**Table 2:** Activated brain regions obtained from the SPM8 (statistical parametric mapping) whole-brain analysis for the following contrasts: starch group less activated than glucose group (Starch < Glucose), and starch group less activated than fructose group (Starch < Fructose). There was no statistical difference between glucose and fructose groups.

		Starch < Glucose				Starch < Fructose			
Brain regions	Hemisphere	x	y	z	Peak T	x	y	z	Peak T
Anterior prefrontal cortex	R					2	28	0	9.43
Dorsolateral prefrontal cortex	R					8	38	6	11.03
Dorsolateral prefrontal cortex	L	-10	34	6	11.01				
Ventral anterior cingulate cortex	L					-2	18	10	4.39
Dorsal anterior cingulate cortex	L	-2	38	2	12.78	0	26	2	5.42
Dorsal anterior cingulate cortex	R					2	26	2	8.83
Insular cortex	R	14	16	12	13.69				
Insular cortex	L	-14	12	14	13.38				
Primary somatosensory cortex	L					-18	20	14	9.63
Anteroventral thalamic nucleus	R					4	8	8	8.66
Mediodorsal thalamic nucleus	L	-2	2	2	13.95				
Somatosensory association cortex	R	20	44	16	8.83				
Parahippocampal cortex	L	-6	-14	10	10.24				

The peak T-value and stereotaxic coordinates (x, y, z in mm) in the CA—CP (*commissura anterior—commissura posterior*) reference plane are indicated for each significant cluster (cluster size ≥5 voxels), for the left (L) and right (R) hemispheres. The P-value uncorrected of the peak of maximal intensity was set at P < 0.001 for all the clusters.

## Brain imaging

**Fig 1:**
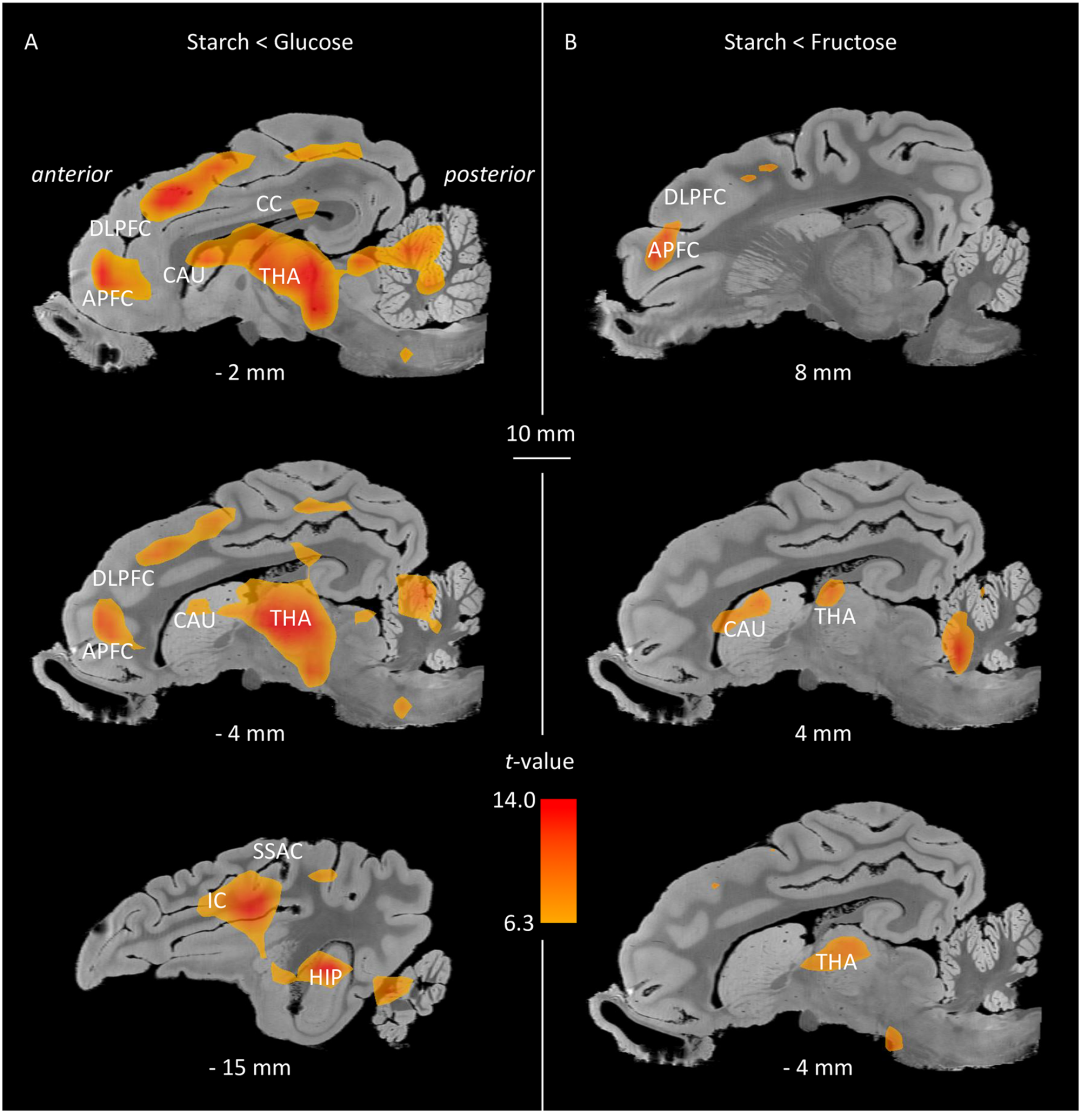
Brain metabolic differences between starch and glucose groups and starch and fructose groups. There was no statistical difference between glucose and fructose groups. Sagittal slices showing some brain structures identified during the whole-brain analyses (*Punc* < 0.001; Threshold *T* = 6.3). (A) Starch group less activated than glucose group, Starch < Glucose (in red); and (B) Starch group less activated than fructose group, Starch < Fructose (in yellow). APFC, anterior prefrontal cortex; DLPFC, dorsolateral prefrontal cortex; IC, insular cortex; CC, cingulate cortex; SSAC, somatosensory association cortex; HIP, hippocampus; THA, thalamus; CAU, caudate nucleus. The x coordinates in the CA-CP (*commissura anterior-commissura posterior*) plane are indicated below the images.

**Fig 2:**
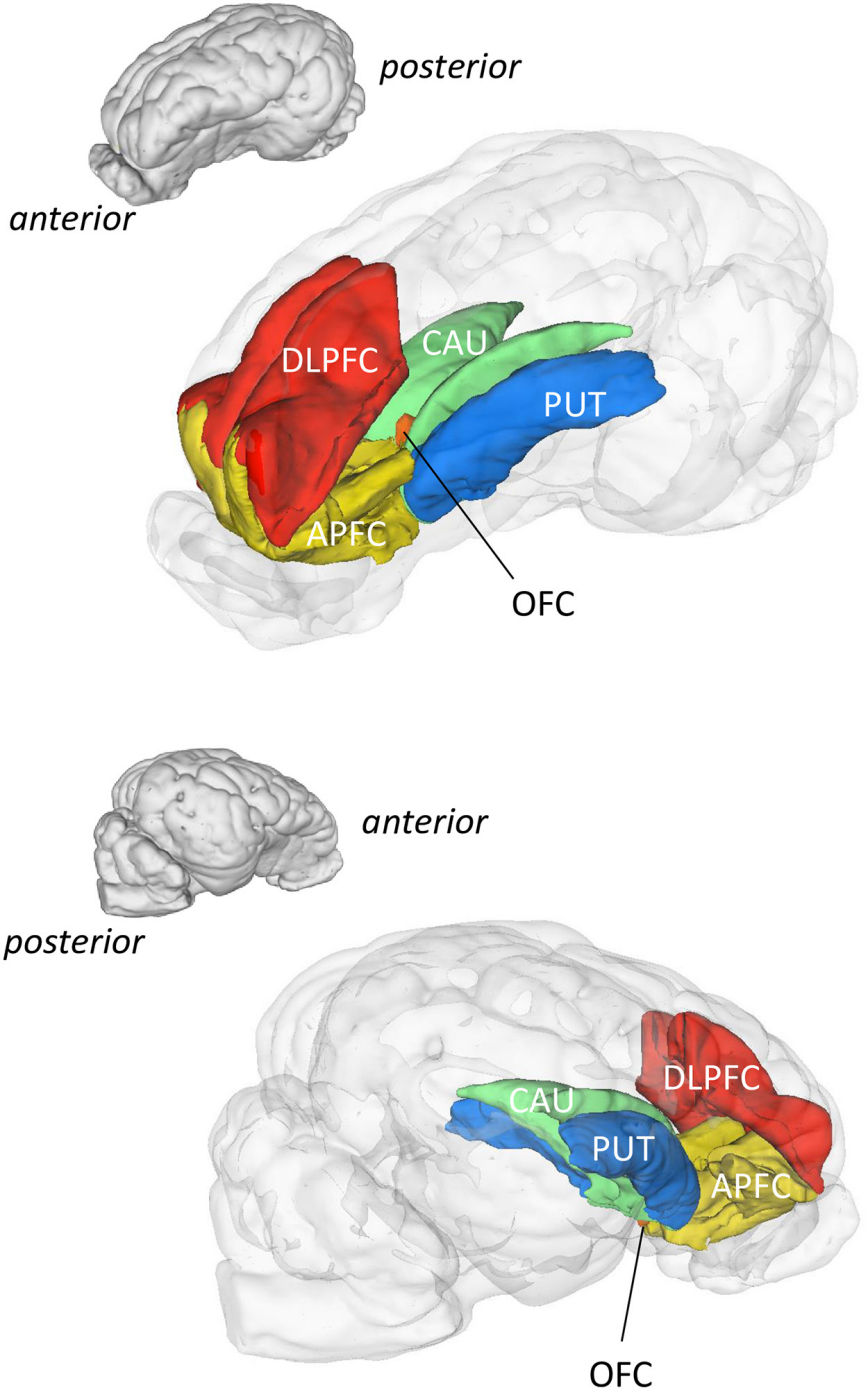
Recap three-dimensional brain models showing the regions of interest (ROI) activated by chronic consumption of sugar-enriched diets. 3D-representations of the ROI that were significantly activated by both glucose and fructose compared to starch group (Starch < Glucose and Starch < Fructose), using the SPM8 (statistical parametric mapping) small volume correction (SVC) analysis (*Punc* < 0.001; Threshold *T* = 6.3). APFC, anterior prefrontal cortex; DLPFC, dorsolateral prefrontal cortex; OFC, orbitofrontal cortex; CAU, caudate nucleus; PUT, putamen. There was no statistical difference between glucose and fructose groups.

The whole-brain analysis performed on the whole brain volume revealed significant differences of activity (basal CGM) between glucose and starch groups, and between fructose and starch groups, in several brain areas including the prefrontal cortex, cingulate cortex, parahippocampal cortex and/or somatosensory regions (Table 2). Three-dimensional representations of several brain structures for which a significant difference on metabolism was found (P_unc_<0.001) in starch *vs*. glucose and in starch *vs*. fructose groups are shown in Fig 1. The results of the SVC analysis in SPM8 are presented in Table 3 and Fig 2. The SVC analysis performed in the APFC, DLPFC, OFC, caudate nucleus, putamen, nucleus accumbens, and globus pallidus revealed an increased basal CGM in several regions for two contrasts (fructose *vs*. starch and glucose *vs*. starch). Brain regions that were found more activated in the fructose group compared to the starch group included (bilaterally) the APFC, DLPFC, OFC, caudate nucleus and putamen. Similar regions were found more activated in the glucose group compared to the starch group: left and right APFC, left and right DLPFC, left OFC, left and right caudate nucleus, left and right putamen, and left globus pallidus. Absolutely no differences were found in basal CGM between fructose and glucose groups, which explains why data from this contrast are not represented in the tables and figures. There were no significant differences between males and ovariectomized females either. The significant threshold was set at P = 0.001 uncorrected.

**Table 3:** Activated brain regions obtained from the SPM8 (statistical parametric mapping) small volume correction (SVC) analysis for the following contrasts: starch group less activated than glucose group (Starch < Glucose), and starch group less activated than fructose group (Starch < Fructose). Brain structures for SVC were chosen on the basis of *a priori* hypotheses. There was no statistical difference between G and F groups.

		Starch < Glucose				Starch < Fructose			
Brain regions	Hemisphere	x	y	z	Peak T	x	y	z	Peak T
Anterior prefrontal cortex	R	0	32	-2	7.26	2	28	0	9.43
Anterior prefrontal cortex	L	-4	38	2	9.78	0	28	-2	8.10
Dorsolateral prefrontal cortex	R	8	30	8	11.83	8	38	6	11.03
Dorsolateral prefrontal cortex	L	-2	28	14	11.78	-2	28	16	7.94
Orbitofrontal cortex	R					2	24	0	5.00
Orbitofrontal cortex	L	0	24	2	4.77	0	22	2	6.51
Caudate nucleus	R	2	20	4	12.00	4	20	6	8.28
Caudate nucleus	L	-2	20	4	10.29	-2	20	6	5.99
Putamen	R	12	14	8	9.34	14	14	4	4.48
Putamen	L	-16	8	4	16.63	-6	24	2	5.03
Nucleus Accumbens	R								
Nucleus Accumbens	L								
Globus pallidus	R								
Globus pallidus	L	-10	12	4	6.50				

The peak T-value and stereotaxic coordinates (x, y, z in mm) in the CA—CP (*commissura anterior—commissura posterior*) reference plane are indicated for each significant cluster (cluster size ≥5 voxels), for the left (L) and right (R) hemispheres. The P-value uncorrected of the peak of maximal intensity was set at P<0.001 for all the clusters.

### Euglycemic-hyperinsulinemic clamp

Euglycemic-hyperinsulinemic clamp data are presented in Table 4. No difference between dietary treatments was found for any of the measured variables. Insulin clamping gave stable plasma glucose concentrations (3.68 ± 0.12 mmol/L; n = 24) in the steady state that were not different from baseline values (3.37 ± 0.11 mmol/L). In contrast, plasma insulin concentrations increased from 18.67 ± 2.06 in the fasting state to 313±7 mUI/L in the steady state (*P* < 0.0001). No difference was found between groups for the insulin sensitivity index (ISI).

**Table 4:** Euglycemic-hyperinsulinemic clamp analysis after 7-month exposure to starch-, glucose- or fructose-containing diets.

	Diets				P-value
	Starch	Glucose	Fructose	SEM	Diets
Weight (kg)	63.5	62.4	64.1	1.5	0.71
Fasting glucose concentration (mmol/L) ^a^	3.4	3.3	3.5	0.2	0.62
Fasting glucose concentration (mg/dL) ^a^	60.9	56.4	60.7	2.6	0.39
Basal insulinemia (μUI/mL) ^b^	21.1	15.1	15.1	2.4	0.14
Steady state insulinemia (μUI/mL) ^c^	303.9	294.6	355.1	32.1	0.35
Steady state GIR (mmol/min) ^d^	0.99	0.96	0.98	0.06	0.84
Steady state GIR (mg/min) ^d^	178.7	171.5	188.3	10.8	0.85
Delta insulinemia (μUI/dl) ^e^	28.3	29.4	33.9	3.2	0.42
GIR adjusted to weight (mg/kg/min)	2.8	2.7	2.8	0.2	0.91
Insulin sensitivity index (mL·min^-1^**·**μUI·kg)	1.3	1.4	1.4	0.1	0.48

^a^Average between plasma glucose concentrations measured at minute -10 and -20 before the beginning of clamp procedure (in mmoL/L and in mg/dL).

^b^ Measured at minute -10 before the beginning of the clamp procedure.

^c^ Measured at minute 170 of the clamp procedure.

^d^ Steady state glucose infusion rate (GIR) at minute 170 of the clamp procedure in mmol/min and mg/min.

^e^ Difference between basal and steady state insulinemia.

SEM, standard error means value for the 3 groups of dietary treatment merged.

## Discussion

This study explored the effects of 7-month intake of three isocaloric diets varying in a substantial fraction of their carbohydrate content: starch, glucose, and fructose on brain activity measured by ^18^FDG-PET. We explored as well the effect of these diets on insulin sensitivity, measured by the euglycemic hyperinsulinemic clamp. Seven-month intake of fructose and glucose diets, compared to starch diet, induced modifications in CGM in several brain regions involved in reward and eating behavior. All diets induced a similar increase in body weight and insulin resistance, indicating that the observed brain changes were independent from body weight gain and peripheral insulin sensitivity.

In the present study, ^18^FDG-PET whole-brain analysis revealed activations in fructose *vs*. starch groups in several brain structures involved in eating behavior, reward and inhibition (Table 2). SVC analyses highlighted bilateral activations, induced by long-term intake of fructose diet, in the APFC, DLPFC, OFC, caudate nucleus and putamen (Table 3). Compared to starch, glucose induced bilateral activations in similar regions including APFC, DLPFC, caudate nucleus, putamen, left OFC and left globus pallidus (Table 3). These greater activations induced by fructose and glucose diets compared to starch, were independent from body weight gain since the starch control group presented the same weight gain evolution than the experimental diet groups. It was previously shown that palatable food receipt leads to greater activation in the anterior cingulate cortex, medial OFC, amygdala, DLPFC and caudate, insula, striatum, and ventromedial prefrontal cortex [8–10, 38], similar to neural activation pattern observed in addictive-like eating behavior [13] and substance dependence [14]. This increased response of reward regions to palatable food, was suggested to increase the perceived reward from food and may increase the risk of overeating and obesity [15]. Therefore, our results indicate that chronic intake of sugars *per se* is able to induce permanent changes in the brain resting state (in the absence of food stimuli) similar to those observed in substance abuse [14] and receipt of highly palatable food in obese subjects. Greater activation of reward-related regions induced by glucose and fructose chronic consumption, compared to starch, may reflect a hyper-functioning condition induced specifically by long-term intake of sugar-containing diets. Consistent to the dynamic vulnerability model [39], it is plausible that our minipigs did not develop a blunted reward response yet, since they were food restricted for the entire experimental diet exposure (7 months) and did not become morbidly obese. The brain metabolism differences observed in our study would rather be in favor of the reward surfeit theory and hypersensitization of the reward circuit, which might precede the reward deficit status that we previously validated in the *ad libitum*-fed obese minipig model [30].

The increased brain activity in the prefrontal and striatal areas might appear contradictory to a previous study published by our group in morbidly obese minipigs [30] and data obtained in obese human patients [16]. To note, the minipigs from the present study were food-restricted and not morbidly obese. Even though all animals significantly gained weight, they were not fed *ad libitum* and did not consume more feed in a group than in another. According to the dynamic vulnerability model of obesity [40], an initial increased response in striatal and somatosensory regions can lead to decreased striatal responses and low D2Rs expression following chronic consumption of palatable food and/or weight gain. As a consequence, our minipig model might confirm that increased striatal glucose metabolism might precede the decreased corticostriatal activity observed in morbidly obese subjects [30], and that chronic consumption of sugar rather than weight gain *per se* has a major role in these dynamic brain anomalies. We can also speculate that, if our minipigs had become morbidly obese after a certain period of dietary exposure, it is likely that they may have passed from a hyper-functioning (the case in the present study) to a hypo-functioning of reward-related brain regions [30]. Only a longitudinal experiment with repeated PET measurements in the same animals, before and after chronic consumption of sweet food, but also before and after the onset of obesity, could answer this question and confirm the hypothesis of a progressive process characterized by the up-regulation of cortico-striatal regions evolving toward a down-regulation of the same areas. Identifying sequential changes in neuronal biomarkers might be very important for clinical purposes, as suggested by Val-Laillet et al. [41].

Bingeing on sugar has been associated with a ‘primed’ mesolimbic dopamine pathway, and recent studies suggest that glucose and fructose engage brain reward and energy-sensing mechanisms in opposing ways and may drive sugar intake through unique neuronal circuits [19, 42]. Interestingly, using a protocol of intermittent access to sugar solutions, Rorabaugh et al. [43] recently demonstrated that sucrose bingeing in rats with access to fructose solution surpassed that of rats with access to glucose solution, showing that fructose and glucose produce divergent degrees of bingeing. Contrary to our initial hypothesis, fructose chronic consumption had the same effects on the basal brain metabolism than glucose, which might also appear in contradiction with previously published papers showing different brain reward responses to these sugars. As previously reminded, a specificity of our study was to homogenize food ingestion between animals and groups, to prevent bingeing on a particular diet. All animals were overfed in comparison to their metabolic needs but were food restricted anyway, which preserved their food motivation. Minipigs from the glucose and fructose groups ingested the same amount of glucose and fructose, respectively (*i*.*e*. approximately 180 g per day), which might explain why the effects on brain metabolism were not different. Perhaps some deleterious effects observed in the literature at the brain level with fructose are simply due to an increased motivation and increased food consumption, and not specifically to fructose in itself. Controlled experimental conditions where different degrees of bingeing amongst diets are prevented might conceal differences between glucose and fructose effects at the brain level. Further studies allowing for *ad libitum* feeding plan would shed light on this question.

It was previously hypothesized that chronic fructose ingestion may alter the activity of brain reward regions, the hedonic response to food and eating behavior probably through a blunted reward response [2] *via* the induction of hyperinsulinemia and peripheral insulin resistance [25, 44, 45]. In the present study, no difference between groups in the steady-state glucose infusion rate and the sensitivity index from euglycemic hyperinsulinemic clamp data was found, indicating the same level of insulin sensitivity in all groups. Using a high insulin bolus at the onset of the clamp (50 mU/kg) as well as a high insulin perfusion rate for the whole clamp (5 mU/kg/min), we found relatively lower values of insulin sensitivity index compared to a previous study in normal-weight Götingen minipigs (2 mU/kg/min) [46]. Another paper using the clamp procedure in Yucatan minipigs obtained GIR values of 14.46 mg/kg/min in normal-weight individuals and 6.41 mg/kg/min in obese minipigs fed a Western diet [47]. Our own minipigs had a GIR value around 3 mg/kg/min. This suggests the presence of insulin resistance in all animals. There are two possible explanations for the absence of difference between groups in the level of insulin sensitivity between groups. First, individuals may adapt to high intakes and long-term intake of these sugars with an increase or loss of detrimental effects [48]. Second, Yucatan minipigs may be resistant to the deleterious metabolic effects of fructose, as was previously shown in Ossabaw minipigs [49]. The effects of lipids and obesity in the development of insulin resistance are well known [50]. Given that our experimental diets had an elevated fat content (2.17% in the standard minipig diet vs. 13.45% in the experimental diets), this high energy content from lipids, together with the overweight in animals, may have contributed to poor insulin sensitivity in animals, rather than the carbohydrate content.

It was previously shown that increased insulin levels provoke an increase in radiotracer assimilation as well as brain glucose utilization [51]. Since no difference between starch, glucose and fructose groups was found for the insulin sensitivity index, the observed differences in CGM between groups of diet measured by ^18^FDG-PET were not related to insulin sensitivity. However, it must be considered that global scaling affects an arbitrary value for the entire brain image, and this process works correctly only in the case where there is no selective uptake on one particular brain region and for one particular treatment. In the case of a high glucose or fructose diet, the selective transport capacities of these sugars across the blood-brain barrier are not equal [52], which might therefore affect uptake in one or several brain regions. In this context, our initial hypothesis postulating that the entire brain receives the same amount of ^18^FDG and that the only factor affecting variability is the injected dose would not be valid. Therefore, further studies should be performed using dynamic PET as well as measurements of sugar transporters in the blood-brain barrier and other brain structures. The dynamic PET would be useful to resolve this problem, where constant plasma glucose measurements are performed to quantitatively measure regional cerebral glucose utilization in order to determine total radiotracer entry into the brain and radiotracer utilization [53].

A PET study showed that morbidly obese humans have lower striatal D2R availability compared to normal-weight subjects, which could predispose them to eat more to compensate for this reward deficit [16]. Previous animal studies also showed profound downregulation of striatal D2R after excessive intake of highly palatable food [54–57], which was not due to weight gain *per se* but rather to the specific consumption of highly palatable food *versus* normal rat chow [58]. Preliminary results, not shown in this paper, suggest different D2R density in the dorsal striatum of Yucatan minipigs exposed to chronic consumption of high-sugar diets compared to control. Further work is needed to confirm these results and investigate whether striatal D2R availability is related to the basal brain metabolism in the minipig model, as it is in humans [16].

Overall, our findings indicate that, in our experimental conditions, *i*.*e*. restriction in net energy intake level, chronic consumption of both glucose- and fructose-containing diets induce alterations of basal brain metabolic activity in structures involved in reward and eating behavior, when compared to a control starch-containing diet, independently from body weight and insulin sensitivity in Yucatan minipigs. These brain abnormalities, reflected by a hyper-functioning of reward-related brain regions, are similar to those found in substance abuse and some obese individuals.
